# Supplementary material for: A nonrandomized open-label phase 2 trial of nonischemic heart preservation for human heart transplantation
Source: Nat Commun. 2020 Jun 12;11:2976. doi: 10.1038/s41467-020-16782-9 (PMC7293246; doi:10.1038/s41467-020-16782-9)
Supplement: Supplementary file 1 — Supplementary Information [file 41467_2020_16782_MOESM1_ESM.pdf]

Supplementary Information for

Nonischemic heart preservation for human heart transplantation: A nonrandomized open-label phase 2 trial

Johan Nilsson*, Victoria Jernryd, Guangqi Qin, MD, Audrius Paskevicius, Carsten Metzsch, Trygve Sjöberg, Stig Steen.

Supplementary Figures

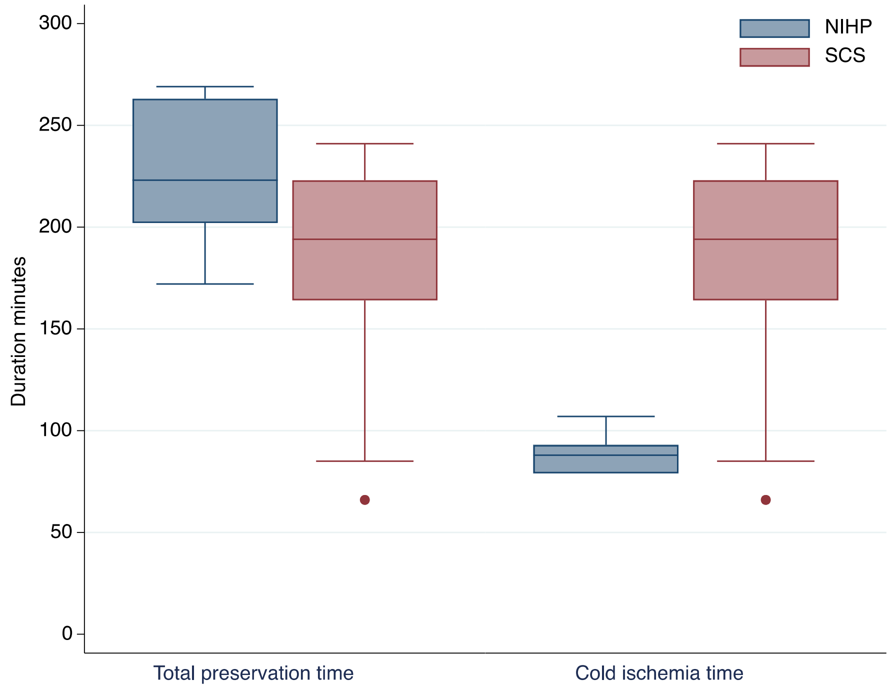

Supplementary Fig. 1 Preservation time according to treatment group. Total preservation time (out-of-body) and total cold ischemia time in the nonischemic heart preservation (NIHP) group (*n* = 6) and the static cold storage (SCS) group (*n* = 25). Data are represented as boxplots where the middle line is the median, the lower and upper hinges correspond to the first and third quartiles, the upper whisker extends from the hinge to the largest value no further than 1.5 × IQR from the hinge (where IQR is the inter-quartile range) and the lower whisker extends from the hinge to the smallest value at most 1.5 × IQR of the hinge, while data beyond the end of the whiskers are outlying points that are plotted individually.

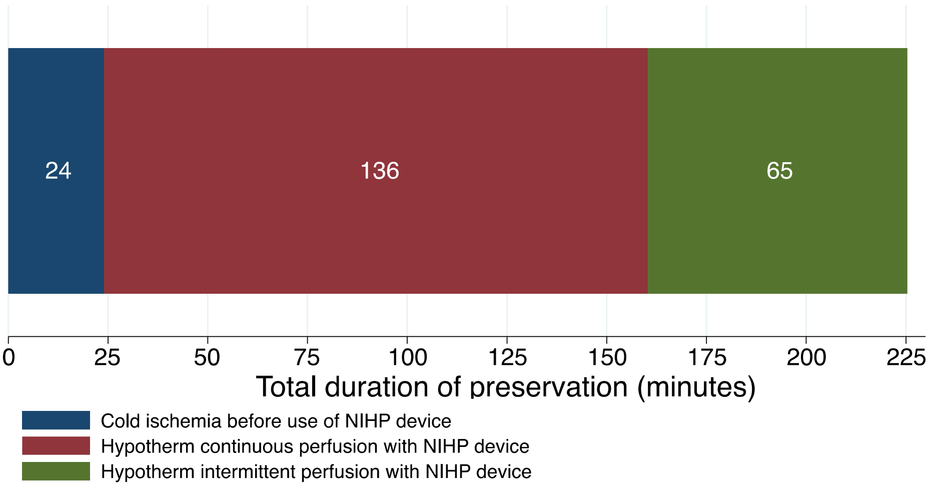

Supplementary Fig. 2 Perfusion time for the nonischemic heart preservation (NIHP) group. The mean cold ischemia time and perfusion time for the 6 donor hearts preserved with the NIHP method. Cold ischemia time consists of the initial retrieval phase (time needed to harvest and implement the heart into the NIHP device).

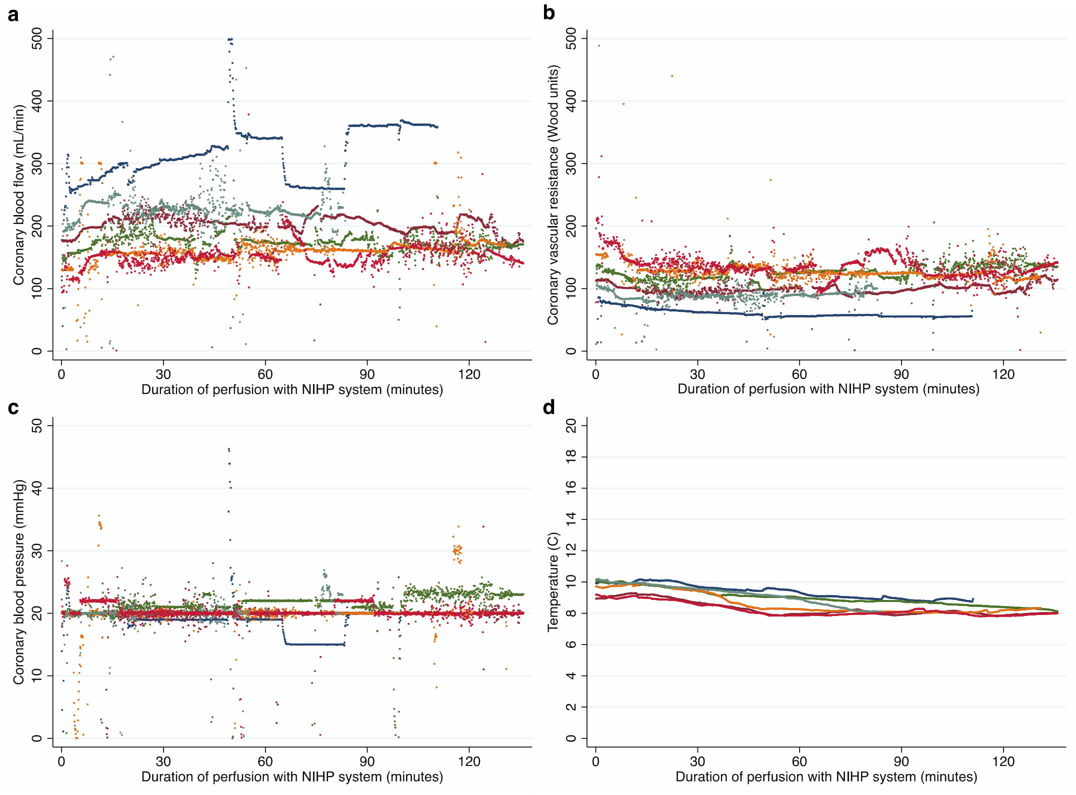

Supplementary Fig. 3 Machine data of nonischemic heart preservation (NIHP). Shown is the coronary blood flow (**a**), the coronary vascular resistance (**b**), the coronary blood pressure (**c**), and the temperature (**d**) for the 6 donor hearts using the NIHP device. Each color represents one patient.

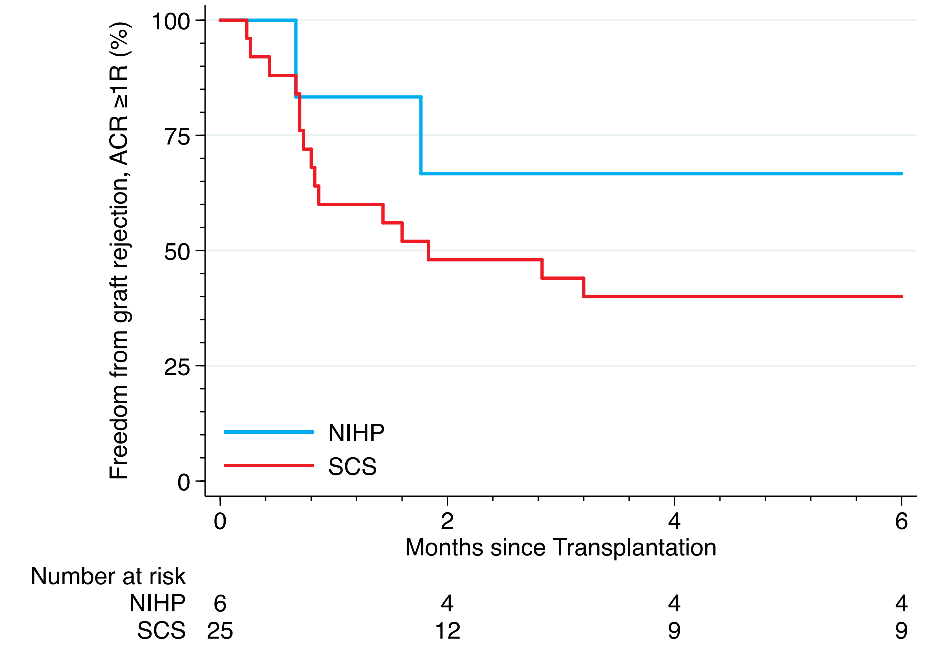

Supplementary Fig. 4 Unadjusted probability of freedom from acute cellular rejection. The Kaplan-Meier plot shows the unadjusted probability of freedom from acute cellular rejection (ACR) ≥1R within 180 days (cyan: NIHP group [n = 6]; red: SCS group [n = 25]). *NIHP* nonischemic heart preservation and *SCS* static cold storage.

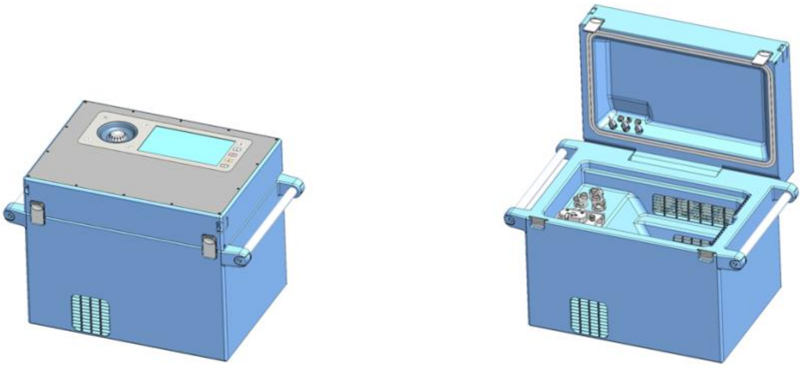

Supplementary Fig. 5 The nonischemic heart preservation (NIHP) device. The pictures show NIHP device: a miniaturized fully automated heart-lung machine housed in a portable device. The equipment consists of a reservoir (not shown in the picture), a pressure-controlled pump, an oxygenator, a leucocyte-arterial filter, a heater-cooler unit, oxygen and carbon dioxide containers, a gas mixer, sensors, and a programmable control system with a backup and a user-friendly interface.

Supplementary Table 1 NIHP procedure and patient outcome data.

Pat. (#)	Flow (mL/min)	Preserv. time (min)	Lac. before perf.	Lac. after perf.	cTnI before perf.	cTnI after perf.	Reper-fusion (min)	RVEF 0-24 h after tx	CI 24 h after tx	Score 24 h after tx	Time on vent. (hours)
1	313	202	.	.	.	.	94	40-50%	2.88	3	54
2	188	263	1.5	1.4	<0.02	<0.02	80	40-50%	3.53	61	36
3	167	269	1.0	1.0	<0.02	<0.02	95	40-50%	3.20	3	22
4	160	219	1.5	1.6	0.08	0.49	119	30-40%	2.25	117	181
5	221	172	1.2	1.5	<0.02	<0.02	83	>50%	4.32	29	12
6	149	227	1.5	1.3	<0.02	<0.02	88	>50%	3.27	40	28

CI cardiac index, *cTnI* cardiac troponin I, *Lac.* lactate, *min* minutes, *mL* milliliter, *Pat.* patient, *perf.* perfusion, *Preserv.* preservation, *RVEF* right ventricular ejection fraction, *Score* inotropic score, and *vent* ventilator, *tx* transplantation, and *NIHP* nonischemic heart preservation.
