# Supplementary material for: A nonrandomized open-label phase 2 trial of nonischemic heart preservation for human heart transplantation
Source: Nat Commun. 2020 Jun 12;11:2976. doi: 10.1038/s41467-020-16782-9 (PMC7293246; doi:10.1038/s41467-020-16782-9)
Supplement: Supplementary file 3 — Reporting Summary [file 41467_2020_16782_MOESM3_ESM.pdf]

nature research

Corresponding author(s): Johan Nilsson (JN)

Last updated by author(s): Apr 1, 2020

Reporting Summary

Nature Research wishes to improve the reproducibility of the work that we publish. This form provides structure for consistency and transparency in reporting. For further information on Nature Research policies, see Authors & Referees and the Editorial Policy Checklist.

Statistics

For all statistical analyses, confirm that the following items are present in the figure legend, table legend, main text, or Methods section.

n/a	Confirmed	
☐	☒	The exact sample size (n) for each experimental group/condition, given as a discrete number and unit of measurement
☒	☐	A statement on whether measurements were taken from distinct samples or whether the same sample was measured repeatedly
☐	☒	The statistical test(s) used AND whether they are one- or two-sided<br>*Only common tests should be described solely by name; describe more complex techniques in the Methods section.*
☐	☒	A description of all covariates tested
☐	☒	A description of any assumptions or corrections, such as tests of normality and adjustment for multiple comparisons
☐	☒	A full description of the statistical parameters including central tendency (e.g. means) or other basic estimates (e.g. regression coefficient) AND variation (e.g. standard deviation) or associated estimates of uncertainty (e.g. confidence intervals)
☐	☒	For null hypothesis testing, the test statistic (e.g. F, t, r) with confidence intervals, effect sizes, degrees of freedom and P value noted<br>*Give P values as exact values whenever suitable.*
☒	☐	For Bayesian analysis, information on the choice of priors and Markov chain Monte Carlo settings
☒	☐	For hierarchical and complex designs, identification of the appropriate level for tests and full reporting of outcomes
☐	☒	Estimates of effect sizes (e.g. Cohen's d, Pearson's r), indicating how they were calculated

Our web collection on statistics for biologists contains articles on many of the points above.

Software and code

Policy information about availability of computer code

Data collection	Data were collected in Microsoft Excel 16.35 (2019 Microsoft Corporation, Redmond, WA).
Data analysis	Statistical analyses were performed using Stata MP statistical package version 16.0 (2019 StataCorp LP, College Station, TX).

For manuscripts utilizing custom algorithms or software that are central to the research but not yet described in published literature, software must be made available to editors/reviewers. We strongly encourage code deposition in a community repository (e.g. GitHub). See the Nature Research guidelines for submitting code & software for further information.

Data

Policy information about availability of data

All manuscripts must include a data availability statement. This statement should provide the following information, where applicable:

- Accession codes, unique identifiers, or web links for publicly available datasets
- A list of figures that have associated raw data
- A description of any restrictions on data availability

Data cannot be shared publicly because of legal and ethical restrictions. Raw data is available to all interested researchers upon from the corresponding author JN. The instruction how to apply and criteria for access to confidential data is available by the Swedish Ethical Review Authority (http://etikprovning.se).

Field-specific reporting

Please select the one below that is the best fit for your research. If you are not sure, read the appropriate sections before making your selection.

☒ Life sciences ☐ Behavioural & social sciences ☐ Ecological, evolutionary & environmental sciences

Life sciences study design

All studies must disclose on these points even when the disclosure is negative.

Sample size	No sample size calculation was performed. A first in man study utilizing a new preservation method should be based on safety principles. Together with the ethical review board we decided that an interim analyzes should be carried out after 3 patients had been transplanted using the NIHP device and a final analysis should be performed after 6 patients have been transplanted. Based on the sample calculation for the randomized study (N=66) a pilot study sample 10% of the larger randomized study should be sufficient according to the literature. The data obtained in this study provide information for the sample size calculations in the randomized study.
Data exclusions	nclusion criteria for donors and recipients were deliberately broad to represent the full spectrum of clinical practice. Organ donors had to be ! 70 years old. Donors were excluded if any of the following criteria were fulfilled: insulin-treated diabetes, significant coronary artery disease, hepatitis B- or C-positive serology, human immunodeficiency virus (HIV)-positive serology, tuberculosis, malignancy, and abnormal ventricular function <45%. All adult (aged "18 years) recipients on our waiting list for heart transplantation were eligible, excluded those with previous solid organ or bone marrow transplantation, grown-ups with congenital heart defects, four or more previous sternotomies, known malignancy, kidney failure (Iohexol plasma clearance at listing <30), liver failure (aspartate aminotransferase, alanine transaminase or total bilirubin >5 times the upper limit of normal, or international normalized ratio >2.0), ongoing septicemia, urgent, and/or systemic inflammatory disorders treated with corticosteroids. The exclusion criteria were pre-established.
Replication	This is the first controlled trial to compare a new state-of-the art machine perfusion technology with static cold storage in human heart transplantation. This findings have confirmed those demonstrated in small scale animal studies. All experiments using the new device was successful. No donor organ was discarded. The biopsies obtained at each visit during follow-up are normally 3-5 pieces from the heart (endomyocardial biopsies) which are sent for histological evaluation. However, they are judged together, and no individual results are presented.
Randomization	This is a nonrandomized trial. Donor hearts were offered to our heart transplant program through Scandiatransplant (http://www.scandiatransplant.org). Assessment of potential donor hearts were based on the usual constellation of clinical factors, including history, coronary angiography, echo assessment, and direct examination of the heart during procurement. The same standard criteria for donor hearts were used for the NIHP group. Transplantation was scheduled in advance when the NIHP method could be used, because the device and team members trained to use the system must be available. Donors and recipients were excluded from the NIHP method only if they met any of the exclusion criteria. All patients eligible for transplantation, but not assigned to the NIHP method who had signed the written informed consent and did not fulfil any exclusion criteria, were included as controls during the study period. Because of the small sample size in both groups, only descriptive statistics were performed.
Blinding	This was an open label study. Due to the nature of the intervention (large device), it was not possible to blind investigators. However, the main end points, PGD and ACR, were assessed blinded.

Reporting for specific materials, systems and methods

We require information from authors about some types of materials, experimental systems and methods used in many studies. Here, indicate whether each material, system or method listed is relevant to your study. If you are not sure if a list item applies to your research, read the appropriate section before selecting a response.

Materials & experimental systems

n/a	Involved in the study	
☒	☐	Antibodies
☒	☐	Eukaryotic cell lines
☒	☐	Palaeontology
☒	☐	Animals and other organisms
☐	☒	Human research participants
☐	☒	Clinical data

Methods

n/a	Involved in the study	
☒	☐	ChIP-seq
☒	☐	Flow cytometry
☒	☐	MRI-based neuroimaging

Human research participants

Policy information about studies involving human research participants

Population characteristics	All adult (aged 18 years or older) recipients on our waiting list for heart transplantation were eligible if they did not fulfil any of the exclusion criteria. Organ donors had to be 70 years or younger and acceptable as a heart donor. Overall, 29% of the donors and 26% of the recipients were women. The median age was 54 years for the donors and 56 years for the recipients. Non-ischemic cardiomyopathy was the most common diagnosis (65%) and 13% had insulin-treated diabetes, 19% peripheral vascular disease and 48% mechanical circulatory support (LVAD). The baseline characteristics were similar between the two groups except for body size. The donor size was similar in the two groups but the NIHP recipients were larger. This resulted in a significant larger unfavorable size mismatch in the NIHP group.
Recruitment	Between April 2, 2017, and September 25, 2018, 42 patients underwent heart transplantation, 11 patients were excluded

Recruitment	because they met one of the exclusions criteria (4), did not provide written informed consent (4), or they needed an urgent transplantation (3). Of the 31 eligible patients the NIHP system was used in six (Fig. 2). Following organ retrieval, all organs were used. Because it was a nonrandomized trial, bias in the selection of both donors and recipients could have affected the results. Another limitation of this study was its unblinded nature. Personnel involved in patient care could have favored the innovative NIHP treatment or favored the established SCS technique, thus leaving the direction of the potential bias open to speculation.
Ethics oversight	The Swedish research ethics committee approved the trial (2016/603).

Note that full information on the approval of the study protocol must also be provided in the manuscript.

Clinical data

Policy information about clinical studies

All manuscripts should comply with the ICMJE guidelines for publication of clinical research and a completed CONSORT checklist must be included with all submissions.

Clinical trial registration	NCT03150147
Study protocol	The study protocol is published as a supplementary information file.
Data collection	Between April 2, 2017, and September 25, 2018, patients accepted for transplantation, who did not fulfil any exclusion criteria, were enrolled in this study after they signed written informed consent. Furthermore, patients on the waiting list were screened for the study (starting April 1, 2017) and those eligible were contacted and included after they signed written informed consent. However, data was collected and analyzed only for patients that reached transplantation. The donor characteristics: blood-group, cause of death, hypertension, diabetes, malignancy, ongoing infection, smoking, need for inotropic support and time on the ventilator were collected when the organ was offered. Recipient data i.e gender, age, blood group, BMI, diagnosis, presence of diabetes, smoking history and medication was registered as well as type of planed procedure at arrival to the hospital. Intra-operative parameters such as time on CPB, reperfusion (time from x-clamp release until end off CPB) and need of inotropic support were collected at operation theatre. Throughout the perfusion process with the NIHP device, the temperature, perfusion flow, and aortic root pressure were continuously monitored with the built-in sensors. Blood samples were retrieved from the reservoir every 30 ±10 minutes for analysis of aB-Lactate, Cardiac troponin I and CK-MB. During the intermittent perfusion (NIHP and SCS group), when the donor heart was implanted, blood samples were retrieved from the coronary sinus in right atrium. aB-Lactate, Cardiac troponin I and CK-MB were measured at the end of preservation and therafter each anastomosis were completed (approximatelly every 15 minutes). Catheter arterial pressure monitoring at rest was performed continuously after transplantation at the ICU. The hemodynamics (mean artery pressure, systolic artery pressure, pulmonary artery pressure, cardiac output, pulmonary vascular resistance, systolic vascular resistance, central venous pressure), use of inotropic support, and I/R biomarker (CK-MB) was registered/collected at 6 ±2h, 12 ±4h, and 24 ±6h after end of preservation. An ECO was performed and registered postoperative day 1 and 7. Biopsies were obtained once a week first month, every second week second up to third month, once a month up to sixth month. The biopsies were sent for histological evaluation and graded according to ISHLT classification 1R, 2R, 3R (Stewart el al 2005). The main end points, PGD and ACR, were blindly assessed.
Outcomes	The primary end-point was a composite of survival free of severe primary graft dysfunction (PGD) at 24 hours, free of extra corporal mechanical support use within 7 days, and free of acute cellular rejection (ACR) >=2R within 180 days. The PGD was assessed according to Kobashigawa, J., et al. Report from a consensus conference on primary graft dysfunction after cardiac transplantation and ACR according to Stewart, S., et al. Revision of the 1990 working formulation for the standardization of nomenclature in the diagnosis of heart rejection. Secondary endpoints included: (1) I/R-tissue injury- difference in cardiac troponin (cTnl) and kinase-muscle/brain (CK-MB) collected at end of preservation, 6±2, 12±4, and 24±6 hours after end of preservation (Triage CARDIO3, Alero with Biosite Triage®MeterPro); (2) immediate graft function as indicated by any one of the following clinical indicators: (i) need for inotropic support (as judged by inotrope score25) in first 6 hours after arrival to ICU; (II) reperfusion time (time from aortic cross-clamp release in the recipient to termination of cardio pulmonary bypass); (iii) left ventricular ejection fraction (EF) <40% on days 1 post-operatively; (iv) right ventricular EF <40% on days 1 post-operatively; (3) postoperative renal function (difference in estimated minimum creatinine clearance within 7 days post-transplant and need for continuous renal replacement therapy within 7 days post-transplant); (4) postoperative liver function, peak aspartate aminotransferase and alanine transaminase within 24 hours post-transplant); (5) postoperative pulmonary function, hour of ventilator requirement; (6) acute cellular rejection >=1R within six months after transplantation; (7) length of stay at the intensive care unit; (8) graft and patient survival at six months. The main end points, PGD and ACR, were blindly assessed. The primary outcome (actuarial survival free of event) was analyzed using the Kaplan–Meier method, and the result is presented with 95% confidence intervals (CIs). The relative risk (RR) and 95% CI were calculated for the outcome variables. The effective size (ES) was used to compare mean values. Data were assumed to have unequal variances and the approximate degrees of freedom was obtained from Welch's formula. Furthermore, continuous variables were log-transformed to fulfil normality assumptions. Because of the small sample size in both groups, only descriptive statistics were performed.
